# User Satisfaction with Child and Adolescent Mental Health Services: Factor Structure of the Experience of Service Questionnaire (ESQ) in Norway and the UK

**DOI:** 10.1007/s10488-025-01436-z

**Published:** 2025-03-29

**Authors:** Yngvild Arnesen, Bjørn Helge Handegård, Børge Mathiassen, Kjersti Lillevoll, Monica Martinussen, Luís Costa da Silva, Jasmine Harju-Seppänen, Abigail Rennick, Jenna Jacob, Julian Edbrooke-Childs

**Affiliations:** 1 Research Group for Clinical Psychology, Department of Psychology, Faculty of Health Sciences, UiT, The Arctic University of Norway, 9037 Tromsø Tromso, Norway; 2 Regional Centre for Child and Youth Mental Health and Child Welfare, Uit the Arctic University of Norway, Tromsø, 9037 Norway; 3 Department of Child and Adolescent Psychiatry, Division of Child and Adolescent Health, The University Hospital of Northern Norway, University Hospital of North Norway, P.O. Box 19, Tromsø, 9038 Norway; 4 Evidence Based Practice Unit, University College of London, Anna Freud Centre, 4-8 Rodney Street, London, N19JH UK

**Keywords:** Psychometric Properties, Bifactor Model, Adolescents, Parents and Carers, Confirmatory Factor Analysis, User Satisfaction

## Abstract

**Background:**

Child and Adolescent Mental Health Services (CAMHS) are expected to track user satisfaction routinely, and to this end, the Experience of Service Questionnaire (ESQ) is increasingly being adopted worldwide. The literature is inconsistent concerning the underlying factor structure of satisfaction measures, and debate is ongoing regarding the evidence of a general satisfaction factor.

**Aim:**

This study aimed to examine the factor structure and dimensionality of the parent/carer and adolescent versions of the ESQ in the UK and Norway.

**Methods:**

Data were retrieved from routine CAMHS clinical practice in the UK and Norway. Three models suggested by the research group were tested through Confirmatory Factor Analysis (CFA) and reliability testing.

**Results:**

A series of CFAs revealed sound psychometric properties of the ESQ in all samples. A bifactor model with a general satisfaction factor and two specific factors of Satisfaction with Care and Satisfaction with Environment fitted the data best, except for the Norwegian adolescent version where a unidimensional model was kept.

**Conclusion:**

The results support the continued use of the ESQ in CAMHS in the UK and Norway and significantly contribute to the literature on user satisfaction by adding evidence of a general satisfaction factor.

## Introduction 

Assessing user satisfaction is increasingly important in evaluating child and adolescent mental health services (CAMHS) worldwide (Ayton et al., 2007; Bear et al., 2022; Lebow, 1982). With the growing acceptance, initiatives regarding accurately measuring the construct of user satisfaction are being acknowledged (Biering, 2010). However, the construct validity of common user satisfaction measures in CAMHS across different countries remain underexplored.

Focusing on understanding families’ experiences and level of satisfaction not only enhances sustained engagement and reduces premature dropouts but is also recognized as a key factor in achieving optimal clinical outcomes (Rickwood et al., 2017). The impact of prioritizing this understanding is evident in clinical practices, at least in the UK. A decade ago, merely 5% of services systematically collected user satisfaction data (Batty et al., 2013; Hall et al., 2014). Recent research, however, indicates a notable shift, with 68–81% of practitioners now routinely incorporating user satisfaction measures in care (Bear et al., 2022). Compared to adult mental health where at least thirty measures of user satisfaction are available (Miglietta et al., 2018), a limited number of user satisfaction measures apply to CAMHS, including tools by Stüntzner-Gibson et al. (1995), Brannan et al. (1996), Garland, Saltzman, et al. (2000), Attride-Stirling (2002), Ayton et al. (2007), Day et al. (2011), and Haugum et al. (2019). Nevertheless, this growing literature emphasizes the value of families’ opinions regarding their satisfaction with services (Ayton et al., 2007; Barber et al., 2006; Biering & Jensen, 2010; Davison et al., 2017; Day et al., 2011; Rickwood et al., 2017; Solberg et al., 2015). Simultaneously, the literature also embodies perspectives representing a common criticism regarding user satisfaction measures often yielding a too general focus on satisfaction and possibly artificially excessive levels of satisfaction (Brannan et al., 1996; Crawford & Kessel, 1999; Kapp et al., 2017; Wolpert et al., 2016). It is important to consider patient-centred measures of user satisfaction that are customized to the unique needs of children, adolescents, and their parents/carers in order to address the complexity involved in CAMHS effectively (Brown et al., 2014). It has been suggested that the lack of measures with such qualities is a barrier to quality improvement in mental health services (Kilbourne et al., 2018).

The Experience of Service Questionnaire (ESQ, formerly known as “CHI-ESQ”) (Attride-Stirling, 2002) is an accessible user satisfaction measure increasingly being adopted in CAMHS in and outside of the UK (Arnesen et al., 2023; Bear et al., 2022; Brown et al., 2014; Bunge et al., 2014; Clark et al., 2018; Derby, 2016; Karagiorga et al., 2024; Khan et al., 2023; Kilburn et al., 2019; Lindevall, 2020; McGrath et al., 2024; Ozer & Halfon, 2024). Originally, this 12-item measure was developed for use across child health care in the UK to verify service delivery anonymously, but nowadays in line with evidence-based practice, the ESQ is recommended for use routinely with other core measures to ensure families’ experiences with the service will be monitored alongside any changes in symptoms or functioning (CORC, 2024b).

Quantitative measures, such as the ESQ, typically reveal overall high satisfaction with services among most families seen at CAMHS (Crawford & Kessel, 1999; Kapp et al., 2017; Wolpert et al., 2016). Patients often provide qualitative descriptions that highlight both the negative and positive aspects of their experiences. However, it’s important to note that these descriptions often contain valuable insights that can help improve patient care (Biering & Jensen, 2010; Crawford & Kessel, 1999). Findings have indicated that families who are satisfied with the service show higher treatment compliance, which in turn enhances both the clinical and social outcomes of care (Fitzpatrick & Hopkins, 1993; Mahin et al., 2004). By promoting satisfaction, services can hopefully reduce the risk of premature termination of treatment or disagreement between families and clinicians regarding care, improving mental health outcomes (Barber et al., 2006; Bjørngaard et al., 2008; Davison et al., 2017; Day et al., 2011; De Haan et al., 2013).

Implementing user satisfaction measures in routine clinical practice faces a persistent challenge: a lack of well-documented measures with adequate psychometric properties (American Educational Research Association, 2014). Many studies rely on reported internal consistency without examining the factor structure (Brown et al., 2014; Young et al., 1995). A review of the literature on quality in satisfaction measures in adult services concludes this problem still endures (Sanchez-Balcells et al., 2018). In a critical review looking into studies of adolescents with CAMHS experience, Biering (2010) delineated three universal factors of satisfaction: satisfaction with the service environment, clinician relationship, and treatment outcome. Biering (2010) underscored the importance of exploring the weak to moderate correlation between child and parent/carer satisfaction and urged researchers to consider previous research when studying satisfaction. Moreover, in the literature on user satisfaction in CAMHS, a pattern emerges where most studies focus on the development of new measures or adaptations of measures from adult mental health services. Acknowledging this, both Biering (2010) and Brown and colleagues (2014) note that research on the ESQ is one of the few satisfaction measures developed in conjunction with prior research on satisfaction.

In a large-scale study in the UK, the original ESQ demonstrated sound psychometric properties for the child, adolescent and parent/carer versions, and it was also found to be a reliable measure of satisfaction that distinguished between services (Brown et al., 2014). Others (Bunge et al., 2014; Davison et al., 2017; Ozer & Halfon, 2024) have corroborated the usefulness of the ESQ in various clinical settings. Originally, a sum-score determined the overall level of satisfaction (Attride-Stirling, 2002; Barber et al., 2006), leaving little room for understanding the drivers of differences in satisfaction. More recently, Brown and colleagues (2014) elaborated on this by revealing a two-factor structure with most items loading on a factor of Satisfaction with the Care provided and the remaining three items loading on Satisfaction with Environment.

Brown and colleagues (2014) identified strong evidence for a two-factor solution with items loading on the factors Satisfaction with Care and Satisfaction with the Environment. They also found common variance between these two factors, tested by exploratory factor analysis, and suggested a strong “halo” effect. This “halo” effect was considered indicative of responses to the ESQ, as with other satisfaction measures, underpinned by a general attribute of satisfaction. Namely that service users’ overall feelings, or general impression, of satisfaction or dissatisfaction characterize their responses to each item in the ESQ. Previous research (Ayton et al., 2007; Brannan et al., 1996; Brown et al., 2014; Garland, Saltzman et al., 2000) suggest evidence of a general satisfaction factor, as specific factors are typically strongly correlated. However, the question of uni- vs. multi-dimensionality has yet to be examined using a bifactor model.

Having access to well-established, standardized and feasible measures is key to comprehensive coverage both clinically and for research purposes in any cultural context (De Vries et al., 2018). As such, translations of existing measures are preferable to the development of language-specific measures (Hafkenscheid et al., 2010). Despite the ESQ being in use across the world, to the best of our knowledge, the only non-English versions of the ESQ psychometrically examined are the Spanish version (Bunge et al., 2014), the Turkish version (Ozer & Halfon, 2024), and the Greek version (Kotsis et al., 2024). The Spanish version of the ESQ was found to be viable for a population selected from private CAMHS in Buenos Aires, but parallel to findings from Sanchez-Balcells et al. (2018) here too results solely relied on the acceptability of reported Cronbach’s Alpha and did not examine for factor structure. The Turkish version affirmed the two-factor solution by Brown et al. (2014). To sustain the growing application of the ESQ in CAMHS worldwide it is crucial to assess whether it accurately captures the intended construct by examining the ESQ factor structure across samples and countries.

## Aims and Objectives

Despite user satisfaction being referred to as a general construct, the only study to have examined the bifactor structure of the ESQ is Kotsis et al. (2024), who found support for a unidimensional factor structure in the Greek version. Given the limited research on the topic, this study aims to add to the understanding of user satisfaction as a construct by further examining the factor structure of the ESQ in Norwegian and UK clinical samples, exploring unidimensional, two-factor, and bifactor solutions. Subsequently, we estimate the reliability of the factor structure with the best fit. Our hypothesis, based on preliminary findings of a general factor for satisfaction as noted by Brown et al. (2014), and influenced by previous research (Attride-Stirling, 2002; Ayton et al., 2007; Barber et al., 2006; Biering, 2010Brown et al., 2014; Day et al., 2011; Garland et al., 2000a, b; Kotsis et al., 2024), is that the bifactor solution would provide the best model fit to the empirical data.

## Methods

### Dataset

The sample for the current paper included families receiving support from CAMHS clinics in Norway and the UK. All included clinics are members of the learning collaborative, the Child Outcomes Research Consortium (http://www.corc.uk.net/). In the included clinics, routine outcome and satisfaction measures are collected as part of larger audits or service evaluations, where both adolescents, parents/carers and clinicians are invited to respond. Both the Norwegian and UK clinics systematically collected the data at two distinct time points using consistent procedures.

Data from the Norwegian sample was collected between December 2013 to December 2016, from one outpatient clinic at CAMHS at the University Hospital of Northern Norway (UNN). ESQ responses from adolescents and parents/carers were digitally collected. A total of 1,205 eligible patients were included.

The UK sample draws on data collected between February 2011 and December 2021 from multiple CAMHS across the UK and has a mix of digital and paper-completed responses, which were submitted annually to CORC’s central research team. A total of 9,761 parents/carers and 10,207 children and young people were included.

## Measures

User satisfaction was assessed both from the adolescent and parent/carer perspective with separate versions of the ESQ, the original ESQ in the UK and the Norwegian translated version in Norway. Currently, no confirmed information has been available regarding the translation procedure for the Norwegian version of the ESQ other than that it is likely that the ESQ was translated to Norwegian in conjunction with a longitudinal epidemiological study in Norway between 2001 and 2012 (Heiervang et al., 2007).

**The Experience of Service Questionnaire (ESQ)** (Attride-Stirling, 2002) is a freely available questionnaire for exploring user satisfaction. Developed through focus groups with children and parents/carers across the child health sector, the ESQ exists in versions tailored to children (9–11), adolescents (12–18) and for parents/carers. The ESQ comprises 12 items rated on a three-point Likert scale: 1= “Certainly true”, 2 = “Partly true”, and 3 = “Not true”, with lower scores indicating higher satisfaction and higher scores indicating greater dissatisfaction. This scoring method aligns with the CORC Snapshot approach (CORC, 2024a). While alternative scoring methods exist, including the CORC + format (1 = “Not true”, 2 = “Partly true”, and 3 = “Certainly true”), which is also required in the NHS Digital Mental Health Services Data Set (MHSDS), and the Item Response Theory (IRT) approach (which codes responses as 0 = “Not true,” 1 = “Partly true,” and 2 = “Certainly true”), we retained the original scoring to maintain consistency with the instruments design as well as the Norwegian dataset, which builds on the CORC Snapshot approach. The ESQ also includes a “Don´t know” response option, which was treated as a non-substantive response and considered missing data in the analysis. Additionally, there are three open-ended questions allowing for free text responses; however, these were not analysed in this study.

Brown and colleagues (2014) found evidence for two highly correlated, but separate, factors named Satisfaction with Care and Satisfaction with Environment, using a two-level latent trait model. The Care factor (Q1-7 and 11–12) has a range from 9 to 27, and the Environment factor (Q8-10) has a range from 3 to 9. Lower scores indicate a higher degree of satisfaction. In the clinic, families respond to the ESQ at evaluation or discharge. Brown et al. (2014) found the ESQ to both be a valid subjective measure of CAMHS experiences and to reliably distinguish between services (Garralda et al., 2000; Goodman, 2001; Gowers et al., 1999; Hanssen-Bauer et al., 2007a; Hanssen-Bauer et al., 2007b; Lundh et al., 2013; Wolpert et al., 2008).

### Procedures

Standard procedures at the clinics include adolescents and parents/carers to be invited to complete the user satisfaction measure, the ESQ, at either discharge or an evaluation point as part of clinical routines. At assessment, demographic data including age and gender was registered. There were no exclusion criteria.

## Ethics

Gathering data in the Norwegian sample was approved by the Information Security Manager at UNN, who acts on behalf of the Norwegian Data Protection Authority. As the data was collected for the purpose of an audit, and only de-identified data was included in the analysis, no written consent was required from the families as procedures of anonymity and safe storage were followed. In the UK, as the study was a secondary analysis of anonymous routinely collected data sample, ethical approval was not required (NHS, 2023).

### Statistical Analysis

Based on previous research, three competing models were tested with confirmatory factor analysis (CFA) using the mean- and variance-adjusted weighted least squares (WLSMV) estimator (Muthén & Muthén, 2018). The UK analyses were performed in RStudio using R version 4.0.3 (RStudio Team, 2020) and for the Norwegian samples we used R version 4.3.2, using the *lavaan* package (Rosseel, 2012). Every model was tested for parents/carers and adolescents in both the UK and Norway. Due to the large sample size, any participants who had not completed all the ESQ items were excluded from the UK analysis. In the Norwegian analysis, pairwise deletion was used. The main aim was to assess the most useful latent structure underlying the 12 items of the ESQ in the following competing models: (1) a unidimensional model where all 12 items load on a general factor of satisfaction (Attride-Stirling, 2002); (2) a two-factor model as suggested by Brown and colleges (2014) with factors for Satisfaction with Care (SWC) (items 1–7, and 11–12) and Satisfaction with Environment (SWE) (item 8–10); (3) a bifactor model with one general factor and two specific factors for SWC (items 1–7, and 11–12) and SWE (items 8–10).

The adequacy of the competing models was evaluated using a range of goodness-of-fit indices, including the Comparative FIT Index (CFI), Tucker-Lewis Index (TLI), root mean square error of approximation (RMSEA), standardized root mean square residual (SRMR) and Chi-square ($\:{\:{\upchi\:}}^{2}$). Criteria were emphasized where CFI and TLI > 0.95, RMSEA < 0.06 (Hu & Bentler, 1999), and SRMR < 0.08 (Asparouhov & Muthén, 2018). Notably, the SRMR outperforms weighted root mean square residual (WRMR) in large samples (DiStefano et al., 2018). Additionally, the $\:{\:{\upchi\:}}^{2}\:$is sensitive to very large samples (like we have for the UK sample), and even trivial misfit may be significant. Therefore, fit interpretations were not solely based on the $\:{\:{\upchi\:}}^{2}$statistic.

The bifactor model was chosen as the superior model (see results below) based on the fit indices. To assess the reliability of the bifactor models, a series of indices were employed using Dueber’s online calculator (Dueber, 2017). As an index of unidimensionality, the Explained Common Variance (ECV) was used (Reise et al., 2010). Internal reliability for factors loading on the general factor was calculated by McDonald´s coefficient omega hierarchical (ω_h_), and McDonald’s omega specific (ω_hs_) was calculated to assess if items of the specific factors (Satisfaction with Care and Satisfaction with Environment) reliably explained residual variances. Higher values of ω_h_ and ω_hs_ indicate greater reliability (Reise et al., 2013). The ECV index represents the variance explained by the general dimension of the total common variance in the model. Notably, there is no “gold standard” ECV value to determine the question on uni-dimensionality (Reise et al., 2013). In a practical guide, Quinn (2014) suggests that an ECV above 0.90 points to a unitary construct being measured and for practical purposes supports the use of an overall score. Conversely, ECV values below 0.70 indicates sub-scores will provide added value over simply reporting an overall score of the construct measured. This suggests values that fall in the grey area between 0.70 and 0.90 need nuanced consideration (Quinn, 2014). In addition, Cronbach’s alpha (α) values were calculated to ascertain the internal consistency of the ESQ scale as a whole, and the two subscales. A value of 0.70 or higher was interpreted as having acceptable internal consistency (European Federation of Psychologists’ Associations, 2013).

## Results

### Descriptive Statistics

#### Norwegian Sample

The total number of participants in the Norwegian sample was 1205 patients. Following the exclusion of individuals with missing ESQ data, the final sample included ESQ responses from 380 parents/carers and 177 individual adolescents.

### UK Sample

The overall UK sample consisted of 214,657 cases. Removing those without ESQ data, and any duplicate patient IDs resulted in a dataset comprising 9,761 parent/carer-reported ESQs and 10,207 adolescent-reported ESQs. The demographic characteristics of the participants included in the analyses are displayed in Table 1.

**Table 1:** Participant demographic variables at referral

	Adolescent				Parent/carer			
	Norway		UK		Norway		UK	
	*n*	*%*	*n*	*%*	*n*	*%*	*n*	*%*
**Gender**								
Male (1)	56	31.6	3817	37.4	213	56.1	4873	49.9
Female (2)	121	68.4	6353	62.2	167	43.9	4880	49.9
*Missing*	0	0	37	0.4	1	0.3	8	< 0.1
**Ethnicity**								
Asian	Not available		422	4.1	Not available		424	4.4
Black			389	3.8			346	3.5
Mixed			427	4.2			435	4.5
Not stated			1128	11.1			1049	10.7
Other			193	1.9			198	2.0
White			6957	68.2			6606	67.7
*Missing*	177	100	691	6.8	381	100	703	7.2
Age of adolescent (mean/ SD)	14.1	2.0	13.5	3.3	10.8	3.4	10.9	4.3
*Missing*	0	0	12	< 0.1	1	0.3	0	0

*N* = 177 Norwegian adolescent sample, *N* = 10,207 UK adolescent sample, *N* = 380 Norwegian parent sample, *N* = 9761 UK parent sample

### Confirmatory Factor Analysis

First, separate confirmatory factor analyses were conducted in each sample to evaluate the fit of the following models: (1) a unidimensional model; (2) a two-factor model with factors for Satisfaction with Care (items 1–7, and 11–12) and Satisfaction with Environment (item 8–10); (3) a bifactor model with one general factor and two specific factors for “Care” (items 1–7, and 11–12) and “Environment” (items 8–10). In three of the four samples, results for the bifactor model exceeded models 1 and 2, suggesting the bifactor model is superior to the alternative models. Details on results for the parent/carer and adolescent versions are presented separately for both Norway and the UK below.

### CFA Parents/Carers

As seen in Table 2, for parents/carers in the Norwegian sample, the model fit considerably improved from model 1 to model 2, and from model 2 to model 3, indicating the Bifactor model had the most acceptable model fit for the Norwegian parent/carer version of the ESQ. For the parent/carer ESQ version in the UK sample, we found similar results to the parallel Norwegian sample, in terms of model fit improving from model 1 to model 2, and from model 2 to model 3 indicating the Bifactor model resulted in the best fit.

**Table 2:** Fit Indices, parent/carer versions of ESQ

Model	Chi-square (df; *p*)	RMSEA (90% CI)	CFI	TLI	SRMR
*Norway*					
1 Unidimensional	327.9 (54; < 0.00005)	0.116 (0.104; 0.128)	0.952	0.941	0.107
2 Two-factor	187.9 (53; < 0.00005)	0.082 (0.069; 0.095)	0.976	0.970	0.083
3 Bifactor	88.4 (42; < 0.00005)	0.054 (0.038; 0.070)	0.992	0.987	0.051
*UK*					
1 Unidimensional	4954.0 (54; < 0.0001)	0.112 (0.109; 0.114)	0.932	0.916	0.040
2 Two-factor	4209.8 (53; < 0.0001)	0.104 (0.101; 0.106)	0.942	0.928	0.031
3 Bifactor	1615.7 (42 < 0.0001)	0.072 (0.069; 0.075)	0.978	0.965	0.019

Norway *N* = 381, UK *N* = 7280. CFI = comparative fit index; TLI = Tucker-Lewis index; RMSEA = root-mean-square error of approximation; SRMR = standardized root mean square residual

While the RMSEA value slightly exceeded the ideal threshold of 0.06, the bifactor model showed the closest approximation compared to alternatives. Both CFI and TLI exceeded the recommended 0.95 threshold, indicating good fit. Additionally, the SRMR supported the bifactor model as the best fit, well below the common cut-off of 0.08. Despite marginal deviation in RMSEA, the bifactor model represented the data more adequately. The bifactor model yielded the most acceptable fit for the UK parent/carer version. Notably, significant chi-square values for both versions suggest misfit, partly due to large sample size in the UK. However, the magnitudes of RMSEA, SRMR, and CFI indicate misfit is not severe for the bifactor model, which was retained for further reliability testing.

### CFA Adolescents

In the Norwegian adolescent sample, small sample size affected estimation (Table 3). Despite this, the unidimensional model (model 1) showed acceptable fit, suggesting limited benefit from adding complexity. Model 3 exhibited an R-square estimate for ESQ item number 1 larger than 1, rendering results unreliable for reliability testing. Non-significant chi-square values were observed for Norwegian adolescents in models 1 and 2. Assessing fit indices criteria, improvements were noted from model 1 to model 2, and model 2 to model 3 for UK adolescents (Table 3). The bifactor model emerged as the most suitable for this sample. Although chi-square values for UK adolescents were highly significant even in model 3, the large sample size partially contributed, with low RMSEA, SRMR, and high CFI indicating manageable misfit.

**Table 3:** Fit Indices, adolescent version of ESQ

Model	Chi-square (df; *p*)	RMSEA (90% CI)	CFI	TLI	SRMR
*Norway*					
1 Unidimensional	58.7 (54; 0.31)	0.022 (0.000; 0.054)	0.998	0.998	0.052
2 Two-factor	51.3 (53; 0.54)	0.000 (0.000; 0.045)	1.000	1.001	0.046
*UK*					
1 Unidimensional	3148.0 (54; <0.0001)	0.091 (0.088; 0.093)	0.939	0.925	0.039
2 Two-factor	2497.7 (53; <0.0001)	0.081 (0.079; 0.084)	0.952	0.940	0.030
3 Bifactor	753.6 (42; <0.0001)	0.049 (0.046; 0.052)	0.986	0.978	0.019

Norway *N* = 177, UK *N* = 6967. CFI = comparative fit index; TLI = Tucker-Lewis index; RMSEA = root-mean-square error of approximation; SRMR = standardized root mean square residual

**Fig. 1:**
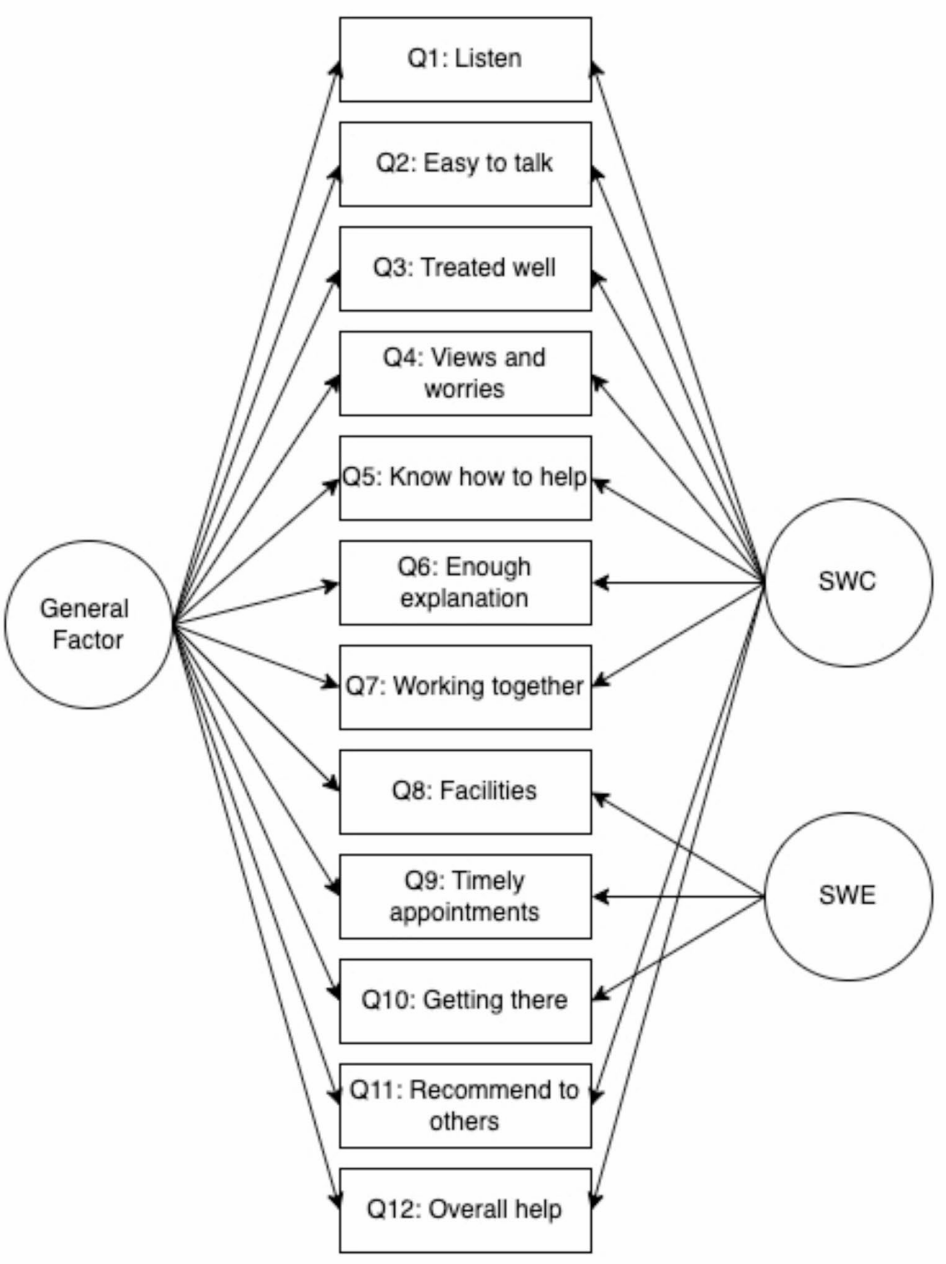
Bifactor model of the ESQ. SWC = Satisfaction with care; SWE = Satisfaction with Environment

To recap, the results from the separate CFAs indicate that the bifactor solution explains the data best in terms of model fit statistics in both countries for parent/carer ESQ and for adolescent ESQ in the UK. For the Norwegian adolescent ESQ, model fit was acceptable for model 1 where all items load on a unidimensional factor of satisfaction, but issues of a limited sample size must be considered. The two-factor solution found by Brown and colleagues (2014) also showed reasonable fit throughout the samples, except for a high RMSEA in the Norwegian parent/carer sample. As the bifactor model (Fig. 1) predominantly demonstrated best statistical model fit, this model was retained to proceed with reliability testing for three of the four samples.

### Reliability

To substantiate the factor structure of the ESQ, we computed model-based reliability indices, including omega hierarchical ($\:{\varpi\:}_{h}$) and omega specific ($\:{\varpi\:}_{hs}$) for each satisfaction factor. These indices, which offer a nuanced assessment of the reliability of the general and specific factors within the bifactor model, are detailed in Table 4. Table 4 shows that for UK parents/carers the general factor explained 88% of the common variance, with an $\:{\varpi\:}_{h}$ of 0.62, suggesting that a substantial proportion of the variance is attributable to the general factor. In the same sample, the specific factor of Satisfaction with Care had only 3% explained common variance. For this subscale, an $\:{\varpi\:}_{hs}$ of 0.002 indicates that the large majority of reliable variance can be attributed to the general factor total score. In contrast, the specific factor Satisfaction with Environment accounted for a larger portion of the common variance at 9%, with a relatively low reliability for the total score for the subscale ($\:{\varpi\:}_{hs}$ = 0.11), capturing a modest amount of unique variance.

For Norwegian parents/carers, the general factor explained 78% of the common variance with an $\:{\varpi\:}_{h}$ of 0.92, demonstrating strong reliability. For Norwegian parents/carers the specific factor Satisfaction with Care explained 9% of the common variance, with an $\:{\varpi\:}_{hs}$ of 0.01 indicating that, similarly to the UK sample, only a very small portion of residual variance of the Satisfaction with Care score is left after partitioning out the variability explained by the total score for the general factor. The specific factor of Satisfaction with Environment explained a larger portion of common variance with 13%, and an $\:{\varpi\:}_{hs}$ of 0.54, suggest that a significant portion of variance is uniquely attributable to this factor.

Similar results were found for the adolescent sample, with the general factor presenting acceptable reliability with most of the variance accounted for by the general factor. As displayed in Table 4, for UK adolescents, the general factor explained 87% of the common variance. The general factor total score accounted for 63% of the test score variance ($\:{\varpi\:}_{h}$ = 0.63), which indicates that a relatively large portion of the reliable variance is accounted for by the general factor. In the same sample, the specific factor of Satisfaction with Care had only 5% explained common variance, with an $\:{\varpi\:}_{hs}$ of 0.01, while the factor Satisfaction with Environment explained 9% of the common variance with an $\:{\varpi\:}_{hs}$ of 0.12.

These results support the presence of a robust general satisfaction factor, with the Satisfaction with Environment subscale also reflecting some unique variance.

**Table 4:** Reliability indices of the ESQ

	UK						Norway		
	Parent version ESQ			Adolescent version ESQ			Parent version ESQ		
	*G*	*SWC*	*SWE*	*G*	*SWC*	*SWE*	*G*	*SWC*	*SWE*
ECV	0.876	0.033	0.092	0.867	0.046	0.087	0.776	0.094	0.130
$\:{{\upvarpi\:}}_{\text{h}}/{{\upvarpi\:}}_{\text{h}\text{s}}$	0.619	0.002	0.114	0.631	0.010	0.117	0.921	0.008	0.538
α	0.94	0.95	0.68	0.92	0.93	0.66	0.88	0.90	0.60

ECV = explained common variance; $\:{\varpi\:}_{h}$= reliability for the general factor test score; $\:{\varpi\:}_{hs}$= the reliability of the specific scores controlling for the general score, based on items relevant to the specific factor; alpha = Cronbach’s alpha; G = General factor; SWC = Satisfaction with Care; SWE = Satisfaction with Environment

## Discussion

In the current study, we assessed the factor structure and psychometric properties of the Experience of Service Questionnaire (ESQ) in Child and Adolescent Mental Health Services (CAMHS) in Norway and the UK, aiming to determine its validity and reliability, particularly examining its factor structure and subscale distinctiveness.

In the confirmatory factor analysis, the bifactor model demonstrated superior fit (according to fit indices as diverse as the CFI, RMSEA and the SRMR) compared to both the unidimensional model and the two-factor model proposed by Brown et al. (2014), which includes the factors Satisfaction with Care (SWC) and Satisfaction with Environment (SWE). Kotsis et al. (2024) recently found support for a unidimensional structure in the Greek version of the ESQ, suggesting that cultural or methodological differences may influence the factor structure across different populations. In addition, Reise et al. (2013) noted that the presence of multidimensionality does not ensure that subscales will yield meaningful and reliable information distinct from the general factor. This is evident in our study, where the SWC subscale score contributes minimal reliable information beyond the general satisfaction score across all three samples examined.

Interestingly, while the SWC subscale, comprising 9 of the 12 items, showed low unique reliability, the SWE subscale displayed higher omega hierarchical values, particularly in the Norwegian parent sample. The three items of the SWE subscale pertaining to the treatment facilities, appointment scheduling, and clinic accessibility may be seen as complex in their measurement of satisfaction. These items could be considered formative indicators of an environmental construct, as they do not necessarily reflect a latent trait of the respondent. Nonetheless, respondents do apply their personal judgment to these aspects of the treatment process, suggesting that a reflective measurement model may still be appropriate.

Given these findings, the utility of the SWC subscale as a distinct measure is questionable. Future research should consider whether modifications to the SWC items could enhance their ability to capture unique variance, or whether alternative methods of assessing specific aspects of care satisfaction are needed.

The results align with a growing body of literature emphasizing the importance of user satisfaction assessment in CAMHS (Athay & Bickman, 2012; Ayton et al., 2007; Nicholas et al., 2017; Rickwood et al., 2017; Simmons et al., 2014). Notably, this study builds on prior research (Attride-Stirling, 2002; Brown et al., 2014; Bunge et al., 2014; Davison et al., 2017; Kotsis et al., 2024) thereby adding insights to the cross-cultural applicability of the ESQ and its validity across diverse clinical settings.

The recognition of a general factor adds depth to the comprehension of user satisfaction in CAMHS. This general factor of user satisfaction suggests an underlying, fundamental dimension contributing to the overall perception of satisfaction, encompassing beyond individual components, that likely considers diverse aspects working together to create a satisfactory experience at CAMHS.

Moreover, identifying a general factor of user satisfaction within CAMHS mirrors already established conceptualizations in psychological research, such as the g-factor of intelligence (Deary et al., 2010; Jensen, 1998) and the p-factor of psychopathology (Caspi et al., 2014; Murray et al., 2016; Patalay et al., 2015). The recognition of this common factor not only aligns with established psychological constructs, but this conceptual alignment also provides a theoretical framework for understanding user satisfaction, fostering a nuanced understanding of the complexities inherent in the evaluation of care in CAMHS.

While highlighting the general factor of user satisfaction took precedence in this study, the specific factors of Satisfaction with Care (SWC) and Satisfaction with Environment (SWE) merit consideration as well. It is worth noting that specific factors were residualized in the bifactor model, indicating what remains after accounting for general satisfaction. However, the very low $\:{\varpi\:}_{hs}$ values for the SWC, suggest that its total score does not capture much unique information beyond what is already explained by the general factor of satisfaction total score. Therefore, the SWC total score seems largely redundant since the general satisfaction factor already accounts for most of the variance in the scores. This redundancy implies that the SWC score may not provide additional insight into the specific aspects of care that are distinct from overall satisfaction.

While interpreting hierarchical omega values, caution is warranted as they serve as an index reflecting the proportion of reliable systematic variance of a subscale after residualization (Reise et al., 2013), and should not be construed as a measure of “reliability” in the traditional sense (Rodriguez et al., 2016). However, our results underscore the unique contribution of the SWE subscale to the overall reliability of the ESQ. Therefore, we advise conservative interpretations, acknowledging that the specific factors may not represent distinct dimensions beyond the general factor. Nonetheless, it is noteworthy that specific factors, especially the SWE subscale, still hold inherent value in assessing user satisfaction in CAMHS, particularly concerning the clinical implications of user satisfaction measures. While our discussion highlights the importance of the “Satisfaction with Environment” (SWE) subscale in enhancing the ESQ’s overall reliability, it’s important to note the predominant emphasis on the Care aspect, evident from the loading of the two general satisfaction items onto the Care subscale. The structural and content differences between the SWC and SWE indicators may contribute to the perceived unique value of the SWE subscale. However, caution is needed in ascribing inherent value to the SWE subscale, as it may also reflect respondent characteristics or subjective perceptions rather than solely objective service quality factors. While these items capture important aspects of service accessibility and logistics, their interpretation may vary depending on individual expectations and contextual influences. Nevertheless, the SWE subscale remains valuable in assessing user satisfaction in CAMHS, particularly concerning its clinical implications.i Additionally, our study found high Cronbach’s alpha values for the general factor in both the UK parent (α = 0.94) and adolescent (α = 0.92) samples, suggesting strong internal consistency under the assumption of unidimensionality. However, the omega hierarchical values were notably lower ($\:{\varpi\:}_{h}$ = 0.62 for parents and $\:{\varpi\:}_{h}$ = 0.63 for adolescents), indicating that the general factor alone does not account for the majority of the variance in the observed scores. This discrepancy highlights the presence of multidimensionality within the scale, and it suggests that specific factors also contribute significantly to the scale’s structure. Therefore, while the scale items are highly interrelated, the interpretation of the general factor should be made with an understanding that it does not fully capture the complexity of user satisfaction as measured by the ESQ.

As the reliability analyses revealed consistent internal consistency for the general factor across both the Norwegian and UK parent/carer, and UK adolescent samples, this suggests the general factor effectively captures the essence of user satisfaction common to both cultural contexts. As robust psychometric properties were revealed in both the Norwegian and UK samples the overall structure and validity of the ESQ appear to transcend cultural boundaries. This suggests the ESQ could serve as a valid measure for assessing user satisfaction in diverse cultural settings.

The implications of our findings for clinical practice in CAMHS are significant. The present study recognizes the multi-faceted nature of user satisfaction, establishing the general factor in user satisfaction as well as specific factors which necessitate targeted interventions but also underscores the interconnectedness of those factors. Especially, the results of our reliability analysis revealed the unique contribution of the subscale SWE to the overall reliability of the ESQ. The omega hierarchical coefficients ($\:{\varpi\:}_{hs})$ linked to this subscale indicate that a significant amount of reliable variance persists even after factoring in the general factor. Conversely, the results imply SWC subscale may not capture a distinct dimension beyond the general factor, as indicated by the omega specific coefficients ($\:{\varpi\:}_{hs})$. As a result, to enhance user satisfaction in CAMHS targeted interventions must be carefully tailored to address specific contexts, with particular emphasis on factors such as the physical environment, accessibility, and scheduling of appointments, which all play pivotal roles in user satisfaction in CAMHS.

Tailoring care interventions to address communication dynamics between clinicians and users, particularly adolescents and their parents, holds promise for enhancing satisfaction. Moreover, acknowledging the impact of the service environment on user experience underscores the need for organizational enhancements within CAMHS. Given the presence of a general satisfaction factor, prioritizing quality improvement emerges as a priority. By combining efforts to address specific concerns with an overarching focus on improving overall satisfaction, CAMHS can make comprehensive advancements. Notably, efforts to enhance satisfaction also play a pivotal role in reducing premature termination of treatment and fostering positive relationships between families and clinicians (Bjørngaard et al., 2008; Hawley & Weisz, 2005). Therefore, routine assessment user satisfaction, encompassing both general satisfaction and specific dimensions, is recommended to ensure that CAMHS continually evolves to meet the diverse needs of its users. In summary, our study not only refines our understanding of user satisfaction in CAMHS but also provides actionable insights for clinicians and service providers to elevate the quality of care and overall user experience.

Acknowledging the strengths of our study, certain limitations must also be considered. Regarding generalizability, certain limitations need consideration. First, the relatively small sample size in the Norwegian adolescent cohort poses challenges to the generalizability of findings. Additionally, we need to exercise caution regarding ethnicity. The UK sample is predominantly White, while ethnicity data is lacking for the Norwegian sample. This skew in representation restricts the generalizability of findings to more diverse populations, meaning caution must be taken when extrapolating conclusions beyond this demographic subset. Additionally, the significant chi-square values in the UK sample warrant cautious interpretation, given the influence of large sample sizes on statistical significance.

Future research endeavours should further examine the ESQ´s applicability across cultural contexts. Since Kotsis et al. (2024) identifyed a unidimensional factor structure in the Greek ESQ, future studies should investigate whether differences in service delivery models, translation procedures, or cultural expectations shape satisfaction ratings. Furthermore, measurement invariance analyses could determine whether the observed differences in factor structures reflect genuine cross-cultural variations or methodological discrepancies in questionnaire adaptation and administration. Also, the limited unique reliability observed in the subscale SWC might benefit from further investigation and possible refinement to better capture the nuanced aspects of satisfaction with care, while enhancing the sensitivity and specificity of the ESQ in assessing user experiences in CAMHS. Additionally, investigations into the responsiveness of the ESQ to specific interventions or changes in service delivery could provide valuable insights for ongoing quality improvement initiatives in CAMHS.

## Conclusion

In summary, this study presents nuanced findings from a psychometric evaluation of the treatment satisfaction measure Experience of Service Questionnaire. A bifactor model demonstrated good model fit, suggesting that a general satisfaction factor underlies the various aspects of treatment satisfaction. However, the analysis also revealed that the Satisfaction with Care (SWC) subscale, which was initially designed to capture specific elements of patient care, exhibited minimal reliable variance when the general factor was accounted for. This indicates that the SWC subscale may not be measuring a distinct dimension of satisfaction beyond what is captured by the general factor.

Moreover, the unexpected high correlations among items within the Satisfaction with Environment (SWE) subscale, particularly those related to the facilitation of treatment, suggest that general respondent satisfaction, potentially influenced by broader life satisfaction or mood, may be confounding the interpretation of the SWE subscale scores. This pattern points to an overarching influence of what we may call respondent disposition, complicating the attribution of satisfaction scores solely to the treatment environment.

These findings underscore the complexity of interpreting subscale scores in the context of treatment satisfaction and highlight the importance of considering general respondent satisfaction when evaluating specific aspects of patient care. The study calls for a careful examination of the factors that influence satisfaction measures and suggests that future research should aim to disentangle the specific contributions of treatment-related factors from the broader psychological state of respondents.

## References

[CR1] American Educational Research Association. (2014). *Standards for Educational and Psychological Testing*. American Educational Research Association.

[CR2] Arnesen, Y., Lillevoll, K. R., & Mathiassen, B. (2023). User satisfaction in child and adolescent mental health service: Comparison of background, clinical and service predictors for adolescent and parent satisfaction. *Health Expectations*, *26*(6), 2608–2619. 10.1111/hex.13861 37650556 10.1111/hex.13861 PMC10632616

[CR3] Asparouhov, T., & Muthén, B. (2018). SRMR in Mplus. In.

[CR4] Athay, M. M., & Bickman, L. (2012). Development and psychometric evaluation of the youth and caregiver service satisfaction scale. *Administration and Policy in Mental Health and Mental Health Services Research*, *39*(1), 71–77. 22407558 10.1007/s10488-012-0407-y PMC3564496

[CR5] Attride-Stirling, J. (2002). *Development of methods to capture users’ views of child and adolescent mental heatlht services in clinical governance reviews project evaluation report*. https://www.corc.uk.net/media/1215/chi_projectevaluationreport.pdf

[CR6] Ayton, A. K., Mooney, M. P., Sillifant, K., Powls, J., & Rasool, H. (2007). The development of the child and adolescent versions of the Verona service satisfaction scale (CAMHSSS). *Social Psychiatry and Psychiatric Epidemiology*, *42*(11), 892–901. 10.1007/s00127-007-0241-9 17700976 10.1007/s00127-007-0241-9

[CR7] Barber, A. J., Tischler, V. A., & Healy, E. (2006). Consumer satisfaction and child behaviour problems in child and adolescent mental health services. *Journal of Child Health Care: For Professionals Working with Children in the Hospital and Community*, *10*(1), 9–21. 10.1177/1367493506060200 16464930 10.1177/1367493506060200

[CR8] Batty, M. J., Moldavsky, M., Foroushani, P. S., Pass, S., Marriott, M., Sayal, K., & Hollis, C. (2013). Implementing routine outcome measures in child and adolescent mental health services: From present to future practice. *Child and Adolescent Mental Health*, *18*(2), 82–87. 10.1111/j.1475-3588.2012.00658.x 32847291 10.1111/j.1475-3588.2012.00658.x

[CR9] Bear, H. A., Dalzell, K., Edbrooke-Childs, J., & Wolpert, M. (2022). Applying behaviour change theory to understand the barriers to implementing routine outcome monitoring. *British Journal of Clinical Psychology*, *61*(3), 557–578. https://bpspsychub.onlinelibrary.wiley.com/doi/pdfdirect/10.1111/bjc.12322?download=true 34319602 10.1111/bjc.12322

[CR10] Biering, P. (2010). Child and adolescent experience of and satisfaction with psychiatric care: A critical review of the research literature. *Journal of Psychiatric and Mental Health Nursing*, *17*(1), 65–72. 10.1111/j.1365-2850.2009.01505.x 20100307 10.1111/j.1365-2850.2009.01505.x

[CR11] Biering, P., & Jensen, V. H. (2010). The concept of patient satisfaction in adolescent psychiatric care: A qualitative study. *Journal of Child and Adolescent Psychiatric Nursing: Official Publication of the Association of Child and Adolescent Psychiatric Nurses, Inc*, *23*(3), 143–150. 10.1111/j.1744-6171.2010.00236.x 20796097 10.1111/j.1744-6171.2010.00236.x

[CR12] Bjørngaard, J. H., Andersson, W., Ose, H. O., S., & Hanssen-Bauer, K. (2008). User satisfaction with child and adolescent mental health services: Impact of the service unit level. *Social Psychiatry and Psychiatric Epidemiology*, *43*(8), 635–641. 10.1007/s00127-008-0347-8 18427704 10.1007/s00127-008-0347-8

[CR13] Brannan, A. M., Sonnichsen, S. E., & Heflinger, C. A. (1996). Measuring satisfaction with children’s mental health services: Validity and reliability of the satisfaction scales. *Evaluation and Program Planning*, *19*(2), 131–141. 10.1016/0149.–7189(96)00004– 3.

[CR14] Brown, A., Ford, T., Deighton, J., & Wolpert, M. (2014). Satisfaction in child and adolescent mental health services: Translating users’ feedback into measurement. *Administration and Policy in Mental Health*, *41*(4), 434–446. 10.1007/s10488-012-0433-9 22829193 10.1007/s10488-012-0433-9

[CR15] Bunge, E. L., Maglio, A. L., Musich, F. M., & Savage, C. (2014). Consumer satisfaction with private child and adolescent mental health services in Buenos Aires. *Children and Youth Services Review*, *47*, 291–296. 10.1016/j.childyouth.2014.10.002

[CR16] Caspi, A., Houts, R. M., Belsky, D. W., Goldman-Mellor, S. J., Harrington, H., Israel, S., & Poulton, R. (2014). The P factor: One general psychopathology factor in the structure of psychiatric disorders? *Clinical Psychological Science*, *2*(2), 119–137. 25360393 10.1177/2167702613497473 PMC4209412

[CR17] Clark, S., Emberly, D., Pajer, K., Delong, E., McWilliam, S., Bagnell, A., & Gardner, W. (2018). Improving access to child and adolescent mental health care: The choice and partnership approach. *J Can Acad Child Adolesc Psychiatry*, *27*(1), 5–14. https://www.ncbi.nlm.nih.gov/pubmed/29375628 29375628 PMC5777686

[CR18] CORC (2024a). *Experience of Service Questionnaire. Scoring and Interpretation*. https://www.corc.uk.net/outcome-experience-measures/experience-of-service-questionnaire-esq/

[CR19] CORC (2024b). *Outcome and Experience Measures. Experience of Service Questionnaire (ESQ)*. Chold Outcomes Reserach Consortium. Retrieved 27.03.24 from https://www.corc.uk.net/outcome-experience-measures/experience-of-service-questionnaire-esq/

[CR20] Crawford, M. J., & Kessel, A. S. (1999). Not listening to patients-the use and misuse of patient satisfaction studies [Peer Reviewed]. *International Journal of Social Psychiatry*, *45*(1). 10.1177/00207640990450010110443244 10443244 10.1177/002076409904500101

[CR21] Davison, J., Zamperoni, V., & Stain, H. J. (2017). Vulnerable young People’s experiences of child and adolescent mental health services. *The Mental Health Review*, *22*(2), 95–110. 10.1108/MHRJ-09-2016-0016/full/html. https://www.emerald.com/insight/content/doi/

[CR22] Day, C., Michelson, D., & Hassan, I. (2011). Child and adolescent service experience (ChASE): Measuring service quality and therapeutic process. *British Journal of Clinical Psychology*, *50*(4), 452–464. 10.1111/j.2044-8260.2011.02008.x 22003953 10.1111/j.2044-8260.2011.02008.x

[CR23] De Haan, A. M., Boon, A. E., De Jong, J. T. V. M., Hoeve, M., & Vermeiren, R. R. J. M. (2013). A meta-analytic review on treatment dropout in child and adolescent outpatient mental health care. *Clinical Psychology Review*, *33*(5), 698–711. 10.1016/j.cpr.2013.04.005 23742782 10.1016/j.cpr.2013.04.005

[CR24] De Vries, P. J., Davids, E. L., Mathews, C., & Aarø, L. E. (2018). Measuring adolescent mental health around the Globe: Psychometric properties of the self-report strengths and difficulties questionnaire in South Africa, and comparison with UK, Australian and Chinese data. *Epidemiology and Psychiatric Sciences*, *27*(4), 369–380. 10.1017/s2045796016001207 28112065 10.1017/S2045796016001207 PMC6998978

[CR25] Deary, I. J., Penke, L., & Johnson, W. (2010). The neuroscience of human intelligence differences. *Nature Reviews Neuroscience*, *11*(3), 201–211. 20145623 10.1038/nrn2793

[CR26] Derby, H. (2016). *Child and Adolescent Mental Health Outcomes Measures: Service Users Satisfaction Survey. Child and Adolescent Mental Health Servide Doha, Qatar*. https://www.hamad.qa/EN/All-Events/5QIMHC/Abstract-Submissions/Abstract_presentations/Pages/Child-and-Adolescent-Mental-Health-Outcomes-Measures-Service-Users-Satisfaction-Survey.aspx.

[CR27] DiStefano, C., Liu, J., Jiang, N., & Shi, D. (2018). Examination of the weighted root mean square residual: Evidence for trustworthiness?? *Structural Equation Modeling: A Multidisciplinary Journal*, *25*(3), 453–466. 10.1080/10705511.2017.1390394

[CR28] Dueber, D. M. (2017). Bifactor Indices Calculator: A Microsoft Excel-based tool to calculate various indices relevant to bifactor CFA models.

[CR29] European Federation of Psychologists’ Associations (2013). EFPA review model for the description and evaluation of psychological and educational tests: Test review form and notes for reviewers, v 4.2.6. *EFPA*.

[CR30] Fitzpatrick, R., & Hopkins, A. (1993). *Measurement of patients’ satisfaction with their care*. Royal College of Physicians of London London.

[CR31] Garland, A. F., Saltzman, M. D., & Aarons, G. A. (2000a). Adolescent satisfaction with mental health services: Development of a multidimensional scale. *Evaluation and Program Planning*, *23*(2), 165–175. 10.1016/s0149-7189(00)00009-4

[CR32] Garland, A. F., Aarons, G. A., Saltzman, M. D., & Kruse, M. I. (2000b). Correlates of adolescents’ satisfaction with mental health services. *Mental Health Services Research*, *2*(3), 127–139. https://www.ncbi.nlm.nih.gov/pubmed/11256722 11256722 10.1023/a:1010137725958

[CR33] Garralda, M. E., Yates, P., & Higginson, I. (2000). Child and adolescent mental health service use. HoNOSCA as an outcome measure. *British Journal of Psychiatry*, *177*, 52–58. https://www.ncbi.nlm.nih.gov/pubmed/10945089 10.1192/bjp.177.1.52 10945089

[CR34] Goodman, R. (2001). Psychometric properties of the strengths and difficulties questionnaire. *Journal of the American Academy of Child & Adolescent Psychiatry*, *40*(11), 1337–1345. 11699809 10.1097/00004583-200111000-00015

[CR35] Gowers, S. G., Harrington, R. C., Whitton, A., Lelliott, P., Beevor, A., Wing, J., & Jezzard, R. (1999). Brief scale for measuring the outcomes of emotional and behavioural disorders in children. Health of the Nation outcome scales for children and adolescents (HoNOSCA). *British Journal of Psychiatry*, *174*, 413–416. https://www.ncbi.nlm.nih.gov/pubmed/10616607 10.1192/bjp.174.5.413 10616607

[CR36] Hafkenscheid, A., Duncan, B. L., & Miller, S. D. (2010). The outcome and session rating scales. A cross-cultural examination of the psychometric properties of the Dutch translation. *Journal of Brief Therapy*, *7*(1), 1–12.

[CR37] Hall, C. L., Moldavsky, M., Taylor, J., Sayal, K., Marriott, M., Batty, M., & Hollis, C. (2014). Implementation of routine outcome measurement in child and adolescent mental health services in the united Kingdom: A critical perspective. *European Child and Adolescent Psychiatry*, *23*(4), 239–242. 23896764 10.1007/s00787-013-0454-2 PMC3973864

[CR38] Hanssen-Bauer, K., Gowers, S., Aalen, O. O., Bilenberg, N., Brann, P., Garralda, E., & Heyerdahl, S. (2007a). Cross-national reliability of clinician-rated outcome measures in child and adolescent mental health services. *Administration and Policy in Mental Health*, *34*(6), 513–518. 10.1007/s10488-007-0135-x 17710527 10.1007/s10488-007-0135-x

[CR39] Hanssen-Bauer, K., Aalen, O. O., Ruud, T., & Heyerdahl, S. (2007b). Inter-rater reliability of clinician-rated outcome measures in child and adolescent mental health services. *Administration and Policy in Mental Health*, *34*(6), 504–512. 10.1007/s10488-007-0134-y 17846880 10.1007/s10488-007-0134-y

[CR40] Haugum, M., Danielsen, K., & Iversen, H. H. (2019). *Development of a questionnaire to measure children’s and adolescents’ experiences with outpatient child and adolescent mental health services*. https://www.fhi.no/globalassets/bilder/rapporter-og-trykksaker/2019/utvikling-av-sporreskjema-for-a-male-barn-og-unges-erfaringer-med-bup-pasopp-rapport-2019.pdf

[CR41] Hawley, K. M., & Weisz, J. R. (2005). Youth versus parent working alliance in usual clinical care: Distinctive associations with retention, satisfaction, and treatment outcome. *Journal of Clinical Child & Adolescent Psychology*, *34*(1), 117–128. 10.1207/s15374424jccp3401_11 15677286 10.1207/s15374424jccp3401_11

[CR42] Heiervang, E., Stormark, K. M., Lundervold, A. J., Heimann, M., Goodman, R., Posserud, M. B., & Gillberg, C. (2007). Psychiatric disorders in Norwegian 8- to 10-year-olds: An epidemiological survey of prevalence, risk factors, And service use. *Journal of the American Academy of Child and Adolescent Psychiatry*, *46*(4), 438–447. 10.1097/chi.0b013e31803062bf 17420678 10.1097/chi.0b013e31803062bf

[CR43] Hu, L., & Bentler, P. M. (1999). Cutoff criteria for fit indexes in covariance structure analysis: Conventional criteria versus new alternatives. *Structural Equation Modeling: A Multidisciplinary Journal*, *6*(1), 1–55.

[CR44] Jensen, A. R. (1998). The factor. *Westport, CT: Prager*.

[CR45] Kapp, C., Perlini, T., Jeanneret, T., Stéphan, P., Rojas-Urrego, A., Macias, M., & Urben, S. (2017). Identifying the determinants of perceived quality in outpatient child and adolescent mental health services from the perspectives of parents and patients. *European Child and Adolescent Psychiatry*. 10.1007/s00787-017-0985-z 28382545 10.1007/s00787-017-0985-z

[CR46] Karagiorga, V. E., Schafer, J. L., Marchionatti, L. E., Caye, A., Serdari, A., Kotsis, K., & Koumoula, A. (2024). Translation and cross-cultural adaptation of seventeen widely-used assessment instruments for child and adolescent mental health in Greece. *Journal of Patient-Reported Outcomes*, *8*(1), 18. 10.1186/s41687-024-00693-0 38345660 10.1186/s41687-024-00693-0 PMC10861406

[CR47] Khan, Y. S., Khoodoruth, M. A. S., Ghaffar, A., Al Khal, A., & Alabdullah, M. (2023). The impact of multisource feedback on continuing medical education, clinical performance and patient experience: Innovation in a child and adolescent mental health service. *Journal of CME*, *12*(1), 2202834. 37123200 10.1080/28338073.2023.2202834 PMC10142306

[CR48] Kilbourne, A. M., Beck, K., Spaeth-Rublee, B., Ramanuj, P., O’Brien, R. W., Tomoyasu, N., & Pincus, H. A. (2018). Measuring and improving the quality of mental health care: A global perspective. *World Psychiatry*, *17*(1), 30–38. 10.1002/wps.20482 29352529 10.1002/wps.20482 PMC5775149

[CR49] Kilburn, T. R., Juul Sørensen, M., Thastum, M., Rapee, R. M., Rask, C. U., Arendt, B., K., & Thomsen, P. H. (2019). Group-based cognitive behavioural therapy for anxiety disorder in children with autism spectrum disorder: A feasibility study. *Nordic Journal of Psychiatry*, *73*(4–5), 273–280. 10.1080/08039488.2019.1622153 31156001 10.1080/08039488.2019.1622153

[CR50] Kotsis, K., Mitropoulou, A., Tzotzi, A., Marchionatti, L. E., Hoffmann, M. S., Schafer, J. L., & Basta, M. (2024). Experience of service questionnaire (ESQ) in children and adolescents: Factor structure, reliability, validity, item parameters and interpretability of the parent version for practical use in Greece. *Journal of Patient-Reported Outcomes*, *8*(1), 128. 39514069 10.1186/s41687-024-00798-6 PMC11549257

[CR51] Lebow, J. (1982). Consumer satisfaction with mental health treatment. *Psychological Bulletin*, *91*(2), 244–259. https://www.ncbi.nlm.nih.gov/pubmed/7071260 7071260

[CR52] Lindevall, O. (2020). *QBUP Årsrapport 2019. Nationelt kvalitetsregister för barn- og ungdomspsykiatri.*

[CR53] Lundh, A., Forsman, M., Serlachius, E., Lichtenstein, P., & Landén, M. (2013). Outcomes of child psychiatric treatment. *Acta Psychiatrica Scand*, *128*(1), 34–44. 10.1111/acps.12043 10.1111/acps.12043 23171318

[CR54] Mahin, A., Attari, A., & Mokhtari, N. (2004). *Compliance and satisfaction in major schizophrenic patients*. European Psychiatry.

[CR55] McGrath, J., Cawley, B., McTiernan, D., Marques, L., Goncerz, E., Heron, E. A.,...& Dowling, B. (2024). Service user satisfaction with care in a specialist service for young people with attention deficit hyperactivity disorder. *Irish Journal of Psychological Medicine*, *41*(1), 46–53. 10.1017/ipm.2022.15 35361298

[CR56] Miglietta, E., Belessiotis-Richards, C., Ruggeri, M., & Priebe, S. (2018). Scales for assessing patient satisfaction with mental health care: A systematic review. *Journal of Psychiatric Research*, *100*, 33–46. 10.1016/j.jpsychires.2018.02.014 29482063 10.1016/j.jpsychires.2018.02.014

[CR57] Murray, A. L., Eisner, M., & Ribeaud, D. (2016). The development of the general factor of psychopathology ‘p factor’through childhood and adolescence. *Journal of Abnormal Child Psychology*, *44*(8), 1573–1586. https://link.springer.com/content/pdf/10.1007/s10802-016-0132-1.pdf 26846993 10.1007/s10802-016-0132-1

[CR58] Muthén, L., & Muthén, B. (2018). Mplus. *The comprehensive modelling program for applied researchers: user’s guide*, *5*.

[CR59] NHS. (2023). *Governance arrangements for research ethics committees: 2020 edition*. N. H. R., & Authority, https://www.hra.nhs.uk/planning-and-improving-research/policies-standards-legislation/governance-arrangement-research-ethics-committees/

[CR60] Nicholas, A., Holloway, E., Telford, N., & Rickwood, D. (2017). Development of the headspace family and friends satisfaction scale: Findings from a pilot study. *Early Interv Psychiatry*. 10.1111/eip.12427 28422429 10.1111/eip.12427

[CR61] Ozer, P. B., & Halfon, S. (2024). Satisfaction in Mental Health Care: Examining Psychometric Properties of Experience of Service Questionnaire in a Turkish Population. 10.1177/13591045241287859 39340220

[CR62] Patalay, P., Fonagy, P., Deighton, J., Belsky, J., Vostanis, P., & Wolpert, M. (2015). A general psychopathology factor in early adolescence. *British Journal of Psychiatry*, *207*(1), 15–22. 10.1192/bjp.bp.114.149591 10.1192/bjp.bp.114.149591 25906794

[CR63] Quinn, H. O. C. (2014). *Bifactor models, explained common variance (ECV), and the usefulness of scores from unidimensional item response theory analyses*.

[CR64] Reise, S. P., Moore, T. M., & Haviland, M. G. (2010). Bifactor models and rotations: Exploring the extent to which multidimensional data yield univocal scale scores. *Journal of Personality Assessment*, *92*(6), 544–559. 10.1080/00223891.2010.496477 20954056 10.1080/00223891.2010.496477 PMC2981404

[CR65] Reise, S. P., Scheines, R., Widaman, K. F., & Haviland, M. G. (2013). Multidimensionality and structural coefficient bias in structural equation modeling: A bifactor perspective. *Educational and Psychological Measurement*, *73*(1), 5–26. 10.1177/0013164412449831

[CR66] Rickwood, D., Nicholas, A., Mazzer, K., Telford, N., Parker, A., Tanti, C., & Simmons, M. (2017). Satisfaction with youth mental health services: Further scale development and findings from headspace - Australia’s National youth mental health foundation. *Early Interv Psychiatry*, *11*(4), 296–305. 10.1111/eip.12248 25996832 10.1111/eip.12248

[CR67] Rodriguez, A., Reise, S. P., & Haviland, M. G. (2016). Evaluating bifactor models: Calculating and interpreting statistical indices. *Psychological Methods*, *21*(2), 137. 26523435 10.1037/met0000045

[CR100] Rosseel, Y. (2012). lavaan: An R package for structural equation modeling. *Journal of Statistical Software, 48*, 1–36.

[CR101] RStudio Team. (2020). RStudio: Integrated Development for R. RStudio, PBC, Boston, MA. http://www.rstudio.com/.

[CR68] Sanchez-Balcells, S., Callarisa Roca, M., Rodriguez-Zunino, N., Puig-Llobet, M., Lluch-Canut, M. T., & Roldan-Merino, J. F. (2018). Psychometric properties of instruments measuring quality and satisfaction in mental health: A systematic review. *Journal of Advanced Nursing*, *74*(11), 2497–2510. 10.1111/jan.13813 30043479 10.1111/jan.13813

[CR69] Simmons, M. B., Parker, A. G., Hetrick, S. E., Telford, N., Bailey, A., & Rickwood, D. (2014). Development of a satisfaction scale for young people attending youth mental health services. *Early Interv Psychiatry*, *8*(4), 382–386. 10.1111/eip.12104 24224930 10.1111/eip.12104

[CR70] Solberg, C., Larsson, B., & Jozefiak, T. (2015). Consumer satisfaction with the child and adolescent mental health service and its association with treatment outcome: A 3-4-year follow-up study. *Nordic Journal of Psychiatry*, *69*(3), 224–232. 10.3109/08039488.2014.971869 25377025 10.3109/08039488.2014.971869

[CR71] Stüntzner-Gibson, D., Koren, P. E., & Dechillo, N. (1995). The youth satisfaction questionnaire: What kids think of services. *Families in Society*, *76*(10), 616–624. 10.1177/104438949507601004

[CR72] Wolpert, M., Fonagy, P., Frederickson, N., Day, C., Rutter, M., Humphrey, N., & Brown, J. (2008). *Review and recommendations for national policy for England for the use of mental health outcome measures with children and young people*. [Book]. Department of Health.

[CR73] Wolpert, M., Jacob, J., Napoleone, E., Whale, A., Calderon, A., & Edbrooke-Childs, J. (2016). *Child- and Parent-reported Outcomes and Experience from Child and Young People’s Mental Health Services 2011–2015*. C. Press.

[CR74] Young, S. C., Nicholson, J., & Davis, M. (1995). An overview of issues in research on consumer satisfaction with child and adolescent mental health services. *Journal of Child and Family Studies*, *4*(2), 219–238. 10.1007/bf02234097

