# Correction To: User Satisfaction with Child and Adolescent Mental Health Services: Factor Structure of the Experience of Service Questionnaire (ESQ) in Norway and the UK

**DOI:** 10.1007/s10488-025-01447-w

**Published:** 2025-05-16

**Authors:** Yngvild Arnesen, Bjørn Helge Handegård, Børge Mathiassen, Kjersti Lillevoll, Monica Martinussen, Luís Costa da Silva, Jasmine Harju-Seppänen, Abigail Rennick, Jenna Jacob, Julian Edbrooke-Childs

**Affiliations:** 1 Research Group for Clinical Psychology, Department of Psychology, Faculty of Health Sciences, UiT, The Arctic University of Norway, 9037 Tromsø Tromso, Norway; 2 Regional Centre for Child and Youth Mental Health and Child Welfare, Uit the Arctic University of Norway, Tromsø, 9037 Norway; 3 Department of Child and Adolescent Psychiatry, Division of Child and Adolescent Health, The University Hospital of Northern Norway, University Hospital of North Norway, P.O. Box 19, Tromsø, 9038 Norway; 4 Evidence Based Practice Unit, University College of London, Anna Freud Centre, 4-8 Rodney Street, London, N19 JH UK

**Correction to: Administration and Policy in Mental Health and Mental Health Services Research**

10.1007/s10488-025-01436-z

In this article, the author’s name “**Luís Costa da Silva**” was incorrectly written as “**Luis da Costa Silva”**.

The correct format for the author’s Given name and Family name is as follows:

Given Name: Luís.

Family name: Costa da Silva.

The original article has been corrected.

